# Self-Recognition and Allorecognition Mechanisms Exert a Significant Influence on the Sex Allocation Patterns of the Pea Aphid

**DOI:** 10.3390/insects15030158

**Published:** 2024-02-27

**Authors:** Yang Li, Shin-Ichi Akimoto, Shi-Yi Jing

**Affiliations:** 1 College of Biology and Agriculture, Zunyi Normal University, Zunyi 563006, China; jingshiyi2@gmail.com; 2 Department of Ecology and Systematics, Graduate School of Agriculture, Hokkaido University, Sapporo 060-8589, Japan; sakimoto3@icloud.com

**Keywords:** *Acyrthosiphon pisum*, sexual generation, sex allocation, reproduction

## Abstract

**Simple Summary:**

This study explores the mechanisms affecting offspring sex ratios in the pea aphid, *Acyrthosiphon pisum* (Harris), a key issue in evolutionary ecology. It tests the impacts of the presence of both the same and different clones, as well as juvenile hormone III (JH III) levels, on offspring sex allocation. Using red and green clones and the agar method, this study set up three initial treatments using sexuparae and tracked daily offspring number and sex. In a mixed-clone treatment, 1 sexupara of the green clone and 20 oviparous females of either red (1G + 20Rov) or green (1G + 20Gov) clone were transferred onto agar leaves, with a control (1G) established for comparison. After the production of sexuparae, JH III doses were applied to them, and hormone titers and sex allocation in offspring were meticulously measured. The results highlighted marked variations in sex allocation, especially an increase in ovipara number in mixed-clone treatment groups. JH III application reversed this effect, indicating that mixed-clone treatment leads to lowered JH titers, which in turn affects sex allocation. This study concludes that *A. pisum* sexuparae can modify offspring sex allocation in response to adjacent clones, showing diverse mechanisms of sex allocation, where JH III plays a critical role.

**Abstract:**

The mechanism controlling sex allocation in the pea aphid, *Acyrthosiphon pisum* (Harris), remains a crucial yet unresolved issue in the field of evolutionary ecology. This study aims to assess the influence of the presence of both self and non-self clones, along with juvenile hormone III (JH III) titer, on the sex allocation of aphid offspring. To this end, red and green clones were utilized as experimental subjects, and the agar method was employed. Initially, three distinct experimental treatments were established using sexuparae, and the daily offspring count and sex allocation in each treatment zone were recorded. Subsequently, an additional experimental condition involving mixed-clone treatments was introduced. This procedure entailed the transfer of a single sexupara and 20 oviparous females from either the red (1G + 20Rov) or green clone (1G + 20Gov) onto a leaf on agar medium. Simultaneously, a control setup with a new sexupara (1G) was established. Three days following sexupara production, a dose of 0, 25, or 50 ng of JH III was applied to the aphids’ abdomens. Subsequently, the titers of JH III in the sexuparae across each treatment group were quantified, and the extent of sex allocation was tallied. The findings demonstrated pronounced disparities in sex allocation among the various treatments and, notably, a substantial increase in the total offspring and oviparous number in the mixed-clone treatment group. The effects of mixed-clone treatment on the sex allocation patterns of the sexupara progeny could be determined by the application of exogenous JH III, indicating that JH may mediate the effects of mixed-clone treatment on sex allocation. Consequently, it can be concluded that *A. pisum* sexuparae possess the capability to modulate their sex allocation in response to the nature of adjacent competitor clones, thereby demonstrating a variety of sex allocation patterns. Throughout this process, JH III plays a pivotal role.

## 1. Introduction

Invertebrates with multivoltine life cycles, such as daphnids and aphids, exhibit polyphenism, which enables them to adapt to seasonally changing environments and predation pressures [1,2,3]. Particularly in aphids, there is a wide variety of morphs, including apterous vs. alate morphs, different apterous morphs on the primary and secondary hosts, and asexual vs. sexual morphs [3,4,5,6]. Most aphid species reproduce asexually during spring and summer under long-day conditions. However, males and oviparous females, the sexual morphs, are induced to copulate and produce fertilized eggs under short-day and low-temperature conditions in autumn [5,7,8,9,10]. Switching between asexual and sexual morphs increases their genetic diversity and is critical to their survival during harsh winters [11,12,13].

Aphids have a seasonal timer that maintains asexual reproduction over several successive generations following hatching, irrespective of physical conditions [7,14,15]. However, if asexual adults are exposed to short-day and low-temperature conditions after the seasonal timer function has expired, the next-generation females (sexuparae) produce sexual morphs [16]. Aphids can measure the scotophase length; lengthening the scotophase contributes to the induction of sexual morphs [17]. In the pea aphid, *A. pisum*, the critical photoperiod inducing male production is longer than that inducing oviparous female production, and ambient temperatures affect these critical photoperiods, which become shorter at higher temperatures [16,18,19,20]. It has been reported that different clones respond differently to the same physical conditions and produce sexual morphs in different proportions, suggesting local maintenance of genetic variation in the population [20,21,22].

*A. pisum* fourth instars or adults (generation 1, G1) that are first exposed to short-day and low-temperature conditions produce viviparous females in the next generation (G2), which produce sexual morphs in G3. Under the longest photoperiod that induces sexual morph production, G2 females produce viviparous females and males 2–3 days later [16,21,22]. Under a shortening photoperiod, G2 females produce fewer viviparous females and more oviparous females, in addition to producing males after that. In some clones, oviparous females and males are produced, respectively, in the first and second halves of the G2 reproductive period [21]. The progeny sequence varies among clones inhabiting the same locality [22]. If the photoperiod remains short, G2 females produce oviparous females in the first half and viviparous females in the second half without producing males. In the wild, males and oviparous females are expected to appear almost simultaneously due to the difference in the critical photoperiod for males and oviparous females and changes in the progeny sequences [16]. A change in the photoperiod directly affects aphids irrespective of the host plant quality [23], inducing sexual morphs by controlling the hormonal titers [24]. Notably, under a short photoperiod, such as in late autumn, only viviparous females, not the sexual morphs, are produced. The production of viviparous females in late autumn is assumed to be an adaptation for passing infrequently occurring mild winters asexually [16].

Juvenile hormones are candidate molecules for the regulation of the reproductive-mode change after photoperiod sensing [25]. This hormone exerts a profound influence on both the reproductive processes and phenotypic traits of insects [24]. Juvenile hormone III (JH III) is the only juvenile hormone found in aphids [26]. The titer of JH III in *A. pisum* diminishes in conditions of low temperatures and short days [24], and treatment with the anti-JH agent precocene induces male production in *Myzus persicae* [27]. At present, there are no studies on the impact of JH III on sex allocation in *A. pisum*.

Although several studies have examined how climatic conditions induce sexual morphs, none have evaluated how biological environments surrounding aphids affect the production of sexual morphs. Previous experiments have reared individual clones separately and exposed them to various photoperiodic and temperature conditions to examine the progeny sequences and sex ratios in a single or a few clones [15,22,28]. However, in the wild, several clones usually coexist on the host plant and produce sexual forms simultaneously [29,30]. If there is a single clone on the host plant in autumn, mating within a clone, i.e., inbreeding, is likely to occur, although winged males could fly to other colonies for outbreeding [30]. In contrast, outbreeding opportunities would be higher if many clones coexisted on the host plant. These breeding patterns could affect the offspring sex ratios [31]. Provided that aphids can discriminate between self and non-self clones, it is likely that aphid clones can adjust their offspring sex ratios or the ratios of sexual and asexual morphs in response to the presence of other clones. Recent studies demonstrated that aphids could discriminate between self and non-self clones and regulate their reproductive rates [32,33]. Therefore, rearing single clones in a container ignores interactions between clones.

The present study examined whether single G2 females alter their offspring sex allocation in response to the presence of another female from their own or a different clone. Using the agar leaf method [34], we transferred one or two G2 females onto pieces of broad bean leaves on an agar medium and counted the numbers of oviparous and viviparous females and males in G3. We paired G2 females from the same or different clones in the two-sexuparae treatment. Furthermore, another type of mixed treatments was devised, each comprising one sexupara of the green clone and twenty oviparous females from either red or green clones. Distinct concentrations of exogenous JH III were then meticulously applied to each treatment. Subsequently, the titers of JH III in the sexuparae across each treatment group were quantified, and the extent of sex allocation was meticulously tallied. The present study aims not only to identify whether aphids possess the ability to detect and respond to the presence of other clones but also to elucidate the underlying evolutionary and physiological factors for such adjustments in their reproductive behaviors.

## 2. Materials and Methods

### 2.1. Insects and Stock Culture

Green-colored (G) and red-colored (R) *A. pisum* clones were used for the experiments, as it was easy to discriminate between them by their body color. These clones were collected from *Vicia cracca* plants in Lanzhou, China (36°03′40″ N, 103°49′55″ E), in 2020. The clones were maintained as monoclonal cultures at a constant temperature (20 °C) and under a 16L:8D photoperiod at an intensity of 5.8–7.3 W/m^2^ in plastic tubes (25 mm in diameter and 100 mm in depth) [34]. The aphids were reared on seedlings of broad bean *Vicia faba*.

### 2.2. Rearing Experiments

The agar cut-leaf method [34] was used to determine whether the interactions between clones affected their offspring sex ratios. The aphids were placed in round plastic containers (100 mm in diameter and 50 mm in height) with lids. Aphids grow and reproduce successfully in the agar cut-leaf rearing system as on broad bean seedlings [34]. We transferred a fourth-instar aphid (G1) from the stock culture onto a cut *V. faba* leaf on agar medium in a plastic container, which was placed in a climatic chamber (GXZ-280; Jiangnan Corporation, Ningbo, China) set to 15 °C, 50–60% relative humidity, and a 10L:14D photoperiod at an intensity of 5.8–7.3 W/m^2^. Under these conditions, three treatments were prepared using G2 fourth instars. In the single-aphid treatment, one green clone nymph (1G) was transferred onto a cut leaf. The two-aphid treatment was created by transferring two green clone nymphs (1G + 1G) onto different leaves. In the mixed-clone treatment, one nymph each from the green and red clones (1G + 1R) was simultaneously transferred onto different leaves. A fresh leaf was added to the medium every four days until the G2 females died. The total number of aphids of each clone per container was counted daily. The number of newborns per day was estimated as the difference between the total numbers of two consecutive days. Dead aphids were removed and excluded from the counting. After G3 aphids became adults, the morphs (winged males and oviparous and viviparous females) were determined microscopically, and their numbers were recorded. Adults containing embryos with compound eyes were considered viviparous females, and those with eggs and no compound eyes were oviparous females. Winged and slender adults were males. All the containers were placed in the same chamber. Ten replicates were prepared for each treatment.

To evaluate the impact of self or non-self clone presence and JH III titer on the sex allocation of aphid offspring, an additional mixed-clone treatment was conducted. This involved transferring a single green G2 fourth instar and 20 oviparous females of the red clone (1G + 20Rov) or green clone (1G + 20Gov) onto a cut leaf on agar medium. In this setup, all resulting nymphs were from the G2 fourth instar, as the oviparous females did not produce nymphs. Concurrently, a new G2 fourth-instar nymph (1G) was placed on a cut leaf. For each treatment, sixty containers were prepared. On the third day post G2 production, 0, 25, or 50 ng of JH III (GC43934; GLPBIO, Montclair, CA, USA), dissolved in 50 nL of acetone, was applied to the aphids’ abdomen, with 20 replicates per concentration. At 10 am three days after application, four G2 individuals were randomly selected from each treatment group, and three replicates were prepared per JH concentration for JH III titer measurement using a JH III ELISA kit (F4802-A; Fankew Corporation, Shanghai, China) following the manufacturer’s instructions. The remaining containers were used to feed the aphids until the G2 individuals perished. The number of G3 morphs and their sex ratio were recorded in all containers, which were placed in a climatic chamber set to 15 °C, 50–60% relative humidity, and a 10L:14D photoperiod at an intensity of 5.8–7.3 W/m^2^.

### 2.3. Statistical Analysis

The effects of the daily mean number of newborn aphids, the total number of aphids, and aphid sex allocation under the various treatments were analyzed using a generalized linear model (GLM). The proliferation rates of the two color types in the mixed-clone treatments were assessed using multivariate analysis of variance (MANOVA). Means of variable pairs were compared using Student’s *t*-test. Statistical analysis was executed using JMP, version 15 (SAS Institute, Cary, NC, USA).

## 3. Results

### 3.1. Effects of Competitive Pressures on Reproduction and Sex Allocation in Sexual Generations of A. pisum

**Figure 1:**
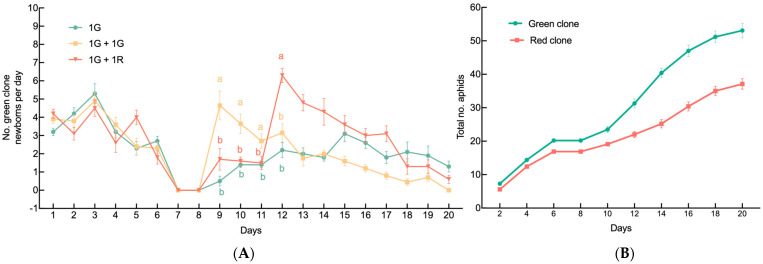
Population growth of colonies founded by a single sexupara. (**A**) Daily production of newborns under each treatment by a sexupara (mean ± SE). (**B**) Populational growth of each clone in 1G + 1R treatment group (mean ± SE). For each treatment, *n* = 10. 1G = one green aphid; 1G + 1G = two green aphids; 1G + 1R = one green aphid coexisting with one red aphid. Different letters indicate significant differences at a significance level of 0.05.

The impacts of neighboring individuals on the offspring production by a single G2 green female are shown in Figure 1A. Over time, neighboring individuals significantly affected the average growth rate of the population produced by G2 green females (GLM, *X*^2^ = 271.44, *df* = 38, *p* < 0.001; Figure 1A). In the 1G + 1G treatment group, the average daily offspring counts were 4.65 ± 0.79, 3.65 ± 0.54, and 2.7 ± 0.4 on days 9, 10, and 11 after the onset of larviposition, respectively. The first number was significantly higher than those in the 1G and 1G + 1R treatment groups, while the other two were similar. However, the average daily offspring count in the 1G + 1R treatment group increased to 6.3 ± 0.4 on day 12, surpassing the other two treatment groups from then onward (Figure 1A). The growth rate of the red clones in the mixed-clone treatment group (1G + 1R) was slower than that of the green clones (MANOVA, F = 30.76, *df* = 2.17,39.1, *p* < 0.001; Figure 1B).

**Figure 2:**
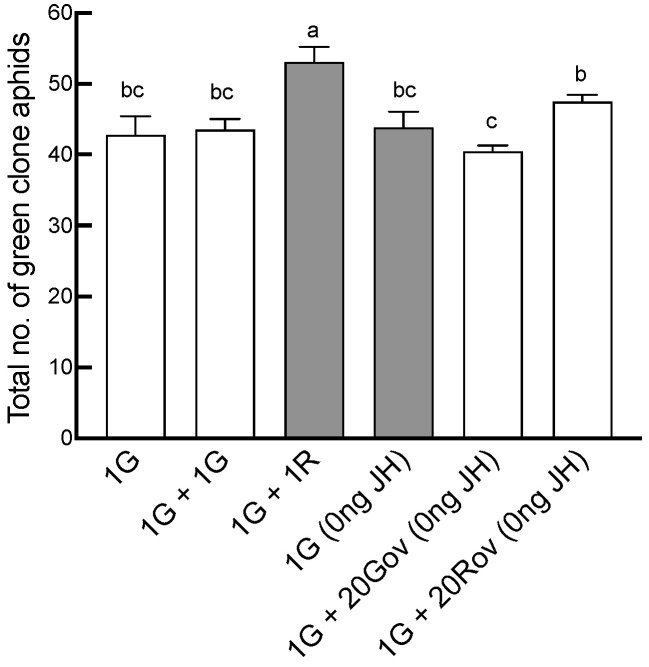
Comparison of the total number of offspring produced by a sexupara under each treatment (mean ± SE). For each treatment, *n* = 8 or 10. 1G = one green aphid; 1G + 1G = two green aphids; 1G + 1R = one green aphid coexisting with one red aphid; 1G (0 ng) = one green aphid with 0 ng of JH III; 1G + 20Gov (0 ng JH) = one green aphid coexisting with 20 green oviparous adults with 0 ng of JH III; 1G + 20Rov (0 ng JH) = one green aphid coexisting with 20 red oviparous adults with 0 ng of JH III. Different letters indicate significant differences at a significance level of 0.05.

**Figure 3:**
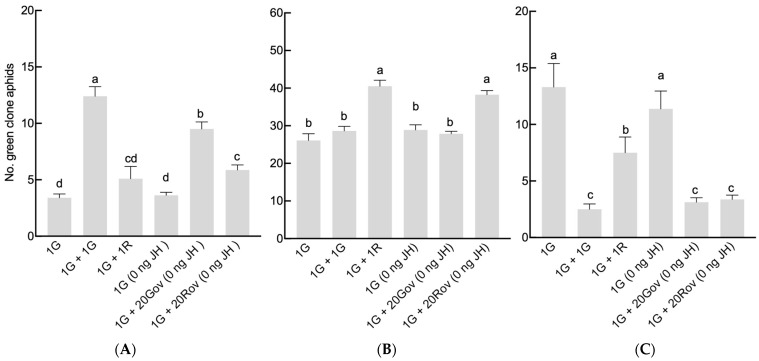
Comparison of the number of offspring produced by a green sexupara under each treatment (mean ± SE). (**A**) The number of males. (**B**) The number of oviparous females. (**C**) The number of viviparous females. For each treatment, *n* = 8 or 10. 1G = one green aphid; 1G + 1G = two green aphids; 1G + 1R = one green aphid coexisting with one red aphid; 1G (0 ng JH) = one green aphid with 0 ng of JH III; 1G + 20Gov (0 ng JH) = one green aphid coexisting with 20 green oviparous adults with 0 ng of JH III; 1G + 20Rov (0 ng JH) = one green aphid coexisting with 20 red oviparous adults with 0 ng of JH III. Different letters indicate significant differences at a significance level of 0.05.

The total number of offspring was significantly affected by neighboring individuals (GLM, *X*^2^ *=* 25.34, *df =* 5, *p* < 0.001; Figure 2). The maximum offspring count occurred in the 1G + 1R treatment group, where green and red clone sexuparae coexisted, averaging 53.1 ± 6.66. This number was significantly greater than those of the other treatment groups (Figure 2). Furthermore, the sex allocation of *A. pisum* was significantly impacted by the type of neighboring individuals (GLM, for males: *X*^2^ *=* 71.9, *df =* 5, *p* < 0.001 (Figure 3A); for oviparous females: *X*^2^ *=* 60, *df =* 5, *p* < 0.001 (Figure 3B); for viviparous females: *X*^2^ *=* 45.44, *df =* 5, *p* < 0.001 (Figure 3C)). In the 1G treatment group, a single green sexupara, the initial aphid, produced an average of 3.4 ± 0.4 males, 26.1 ± 1.77 oviparous females, and 13.3 ± 6.62 viviparous females. The highest count of males (12.40 ± 0.85) was observed in the 1G + 1G treatment group. In the 1G + 20Gov (0 ng JH) treatment group, where the green clone coexisted with a high-density population of oviparous females of the same clone, the male count (9.50 ± 0.63) was slightly lower than in the 1G + 1G treatment group, yet significantly higher than that in the other treatment groups (Figure 3A). Conversely, the male count in the 1G + 1R and 1G treatment groups remained similar. However, the numbers of oviparous females in the 1G + 1R and 1G + 20Rov treatment groups were significantly larger than in the other treatment groups, with no notable difference between them (Figure 3B). The number of viviparous females of the green clone significantly decreased with the increase in the initial sexuparae population density or when coexisting with another clone (Figure 3C). There was no difference in sex allocation between 1G and 1G (0 ng JH).

### 3.2. Effect of Exogenous JH III on Sex Allocation

**Figure 4:**
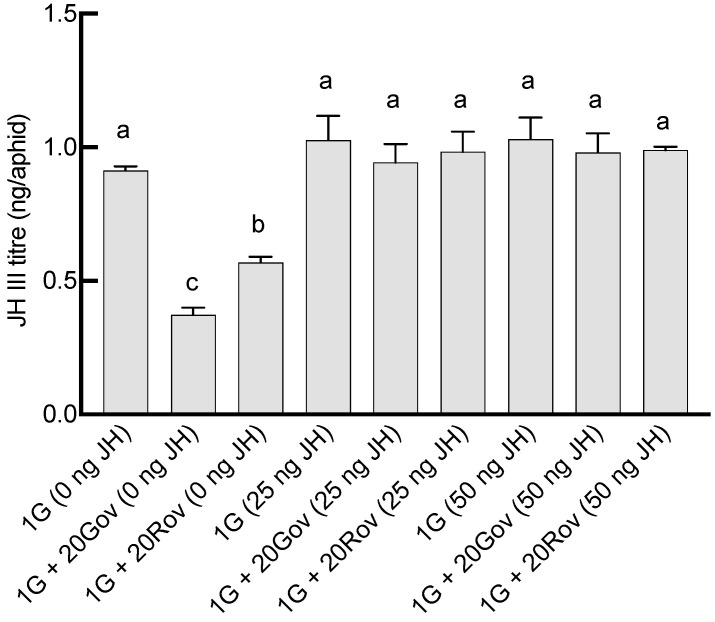
The impact of varying doses of JH III application on the JH III titers in green *A. pisum* sexuparae on the sixth day of oviposition (mean ± SE). For each treatment, *n* = 3. 1G (0 ng JH) = one green aphid with 0 ng of JH III; 1G + 20Gov (0 ng JH) = one green aphid coexisting with 20 green oviparous adults with 0 ng of JH III; 1G + 20Rov (0 ng JH) = one green aphid coexisting with 20 red oviparous adults with 0 ng of JH III; 1G (25 ng JH) = one green aphid with 25 ng of JH III; 1G + 20Gov (25 ng JH) = one green aphid coexisting with 20 green oviparous adults with 25 ng of JH III; 1G + 20Rov (25 ng JH) = one green aphid coexisting with 20 red oviparous adults with 25 ng of JH III; 1G (50 ng JH) = one green aphid with 50 ng of JH III; 1G + 20Gov (50 ng JH) = one green aphid coexisting with 20 green oviparous adults with 50 ng of JH III; 1G + 20Rov (50 ng JH) = one green aphid coexisting with 20 red oviparous adults with 50 ng of JH III. Different letters indicate significant differences at a significance level of 0.05.

**Figure 5:**
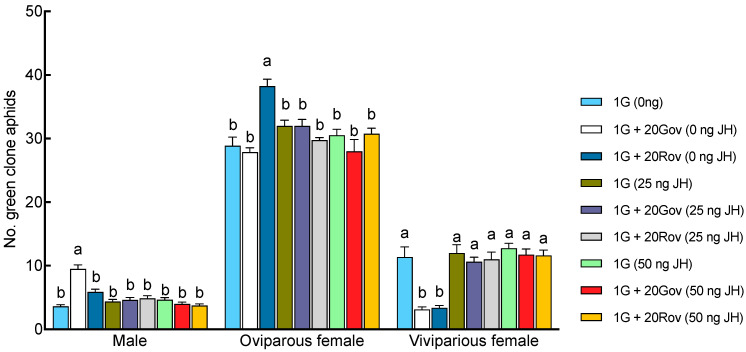
Effects of different JH III applications on sexual allocation of *A. pisum* sexuparae (mean ± SE). For each treatment, *n* = 8. 1G (0 ng JH) = one green aphid with 0 ng of JH III; 1G + 20Gov (0 ng JH) = one green aphid coexisting with 20 green oviparous adults with 0 ng of JH III; 1G + 20Rov (0 ng JH) = one green aphid coexisting with 20 red oviparous adults with 0 ng of JH III; 1G (25 ng JH) = one green aphid with 25 ng of JH III; 1G + 20Gov (25 ng JH) = one green aphid coexisting with 20 green oviparous adults with 25 ng of JH III; 1G + 20Rov (25 ng JH) = one green aphid coexisting with 20 red oviparous adults with 25 ng of JH III; 1G (50 ng JH) = one green aphid with 50 ng of JH III; 1G + 20Gov (50 ng JH) = one green aphid coexisting with 20 green oviparous adults with 50 ng of JH III; 1G + 20Rov (50 ng JH) = one green aphid coexisting with 20 red oviparous adults with 50 ng of JH III. Different letters indicate significant differences at a significance level of 0.05.

The JH III titers on day 6 post sexupara production were 0.57 ± 0.02 ng/aphid in the 1G + 20Rov (0 ng JH) treatment group and 0.37 ± 0.05 ng/aphid in the 1G + 20Gov (0 ng JH) treatment group (Figure 4). In the 1G (0 ng JH) treatment group, the JH III titer was 0.91 ± 0.03 ng/aphid. The titers on day 6 post parturition in both the 1G + 20Gov and 1G + 20Rov treatment groups were significantly lower for sexuparae, with notable differences between them (Figure 4). When exogenous juvenile-preserving hormone was applied, the titers in all treatment groups were similar to that in the control treatment group (1G, 0 ng JH; Figure 4). Significant interactions between high-density population types and the amount of exogenous JH III applied on sexual allocation were found (GLM, for males: *X*^2^ = 63.56, *df* = 4, *p* < 0.001; for oviparous females: *X*^2^ = 38.65, *df* = 4, *p* < 0.001; for viviparous females: *X*^2^ = 22.46, *df* = 4, *p* < 0.001). Under experimental conditions devoid of JH III supplementation, comparisons with the 1G (0 ng JH) treatment group revealed a pronounced decrease in the viviparous female population across the 1G + 20Gov (0 ng JH) and 1G + 20Rov (0 ng JH) treatment groups; simultaneously, the 1G + 20Gov treatment group experienced a substantial escalation in male numbers, while the 1G + 20Rov treatment group witnessed a marked augmentation in oviparous female numbers (Figure 5). Post application of JH III, there was a discernible increase in viviparous females within the 1G + 20Gov and 1G + 20Rov treatment groups, coupled with a decrease in males in the 1G + 20Gov treatment group and oviparous females in the 1G + 20Rov treatment group; the sex allocation pattern of the sexuparae offspring was similar to that of the 1G (0 ng JH) treatment group (Figure 5).

## 4. Discussion

In this study, the agar method was used for the experiments. Rearing *A. pisum* by this method does not differ in individual size and developmental time from rearing this species on *V. faba* seedlings [34]. This rearing technique has been applied in studies related to *A. pisum* and other insects [33,35,36,37,38].

Many studies have found that the offspring sex ratio of a single *A. pisum* was skewed toward females [22,28], as found in the present study. In the 1G + 1G treatment group, the initial number of sexuparae was altered. Although the total number of G3 remained similar to that in the 1G treatment group, the number of male aphids increased significantly, while the number of viviparous females decreased significantly relative to 1G. When resources are sufficient, the density-related competition effect does not appear. However, when resources are limited, the density affects the population development process [39]. In the 1G treatment, where resources were relatively abundant, sexuparae invested part of their resources in fetal aphids capable of solitary reproduction, which allows for a more rapid and direct increase in the number of individuals of the same clone while avoiding additional costs associated with sexual reproduction [12]. Julliard [40] suggests that in environments of suboptimal quality, if there is a gender-based distinction in dispersal tendencies, prioritizing the production of the gender that is more inclined to disperse could be a beneficial adaptation, and sex allocation could be affected by competition for limited resources among individuals of the same population. To reduce competition, individuals may produce more offspring of the sex that can migrate. In the 1G + 1G treatment, due to the increase in the number of aphids in the medium, available resources were lower than in the 1G treatment, resulting in increased intraclonal competition for resources (e.g., food and space). The green clone males in this study were winged. In the wild, the coexistence of co-clonal sexuparae on the host plant is very common. Sexuparae transfer the resources that should have been invested in viviparous females to produce male aphids. This not only improves the reproductive success of the co-clonal population and reduces competition between co-clonal oviparous females but also allows winged males to continue reproducing after leaving undesirable environments when resources are limited. Therefore, in the 1G + 1G treatment group, the clones producing more male offspring was advantageous for population sustainability. The mode of sex allocation of green clones in the 1G + 1G treatment group under conditions of intense resource competition supports Julliard’s suggestion [40].

Red and green *A. pisum* clones show significant differences in their biological characteristics [41,42,43]. In the 1G + 1R treatment, the green clone altered its reproductive strategy due to competition with the red clones. While the number of male offspring did not change, the number of viviparous females decreased significantly, whereas the number of sexuparae and the total number of offspring increased significantly compared with the 1G treatment. Offspring sex ratios of aphids in periodic solitary reproduction might be affected by competition among stem mothers [44] and by the investing of more resources in oviparous females when a green clone encounters a non-self clone. The number of pedigree stem mothers produced increases, placing them in a favorable position when faced with a non-pedigree competitor in the early spring of the following year. Consequently, the genes of sexually reproducing mothers are better disseminated in the population [45]. Our previous study showed intense competition when green *A. pisum* clones coexisted with yellow clones [33]. Reproduction of *A. pisum* was inhibited when placed in high-density populations of non-self clones. Parasite pressure could affect aphid reproduction [46]. There is a ‘priority effect’ during aphid population formation. Colonies with higher fecundity or the first to invade the host could occupy favorable space and resources, creating an absolute advantage over their competitors [47]. In the 1G + 1R treatment group, the growth rate of the red clone was significantly slower than that of the green clone, which dominated by day 4 after the start of competition. Both clones stopped reproducing on day 6. After the resumption of reproduction and until the death of the sexuparae, the green clone remained dominant in the number of G3. It is hypothesized that when faced with a less competitive non-self clone, the green clone strengthens its hold on resources by increasing the number of sexuparae. The competition from non-native clones seems to significantly enhance the overall sexual reproductive capacity of the green clone. In another study, analyzing competition experiments between *A. pisum* and *Megoura.viciae*, we found that *A. pisum*, obtaining competitive victories, reproduced at a higher rate than under intraclonal competition (unpublished results). Similar phenomena have been observed in the plant kingdom. For example, when sowing a mixture of *Plantago asiatica* and *Trifolium repens*, the germination synchronization rate of *P. asiatica* planted adjacent to each other was higher than that in contiguous planting, suggesting possible communication between plantain seeds that promotes germination synchronization [48].

Two factors could be assumed to influence the sexual allocation of aphids: first, how well they mate within the colony; second, the timing of investment in offspring [49]. *A. pisum* sexuparae pause reproducing for about 2–3 days after producing offspring for 6 days. On days 9–11, the average daily offspring count was significantly higher in the 1G + 1G treatment group than in the 1G or 1G + 1R treatment group, maintaining this advantage. However, from day 12 onwards, the number of produced offspring in the 1G + 1R treatment group exceeded that of the other two treatment groups. It was observed that all treatment groups produced mainly males on days 9–11; however, from day 12 onwards, the 1G + 1R treatment group produced mainly oviparous females. It is known that the cost of producing oviparous females in *Prociphilus oriens* is 1.85 times higher than that of producing males [50], which may imply that the cost of producing males is also lower than that for making sexual females for *A. pisum* sexuparae. In the 1G + 1R treatment, sexuparae devoted more resources to oviparous females, resulting in their increase in numbers. This surge in numbers occurred on day 12 due to the relatively high cost required for producing oviparous females and the relatively long time it takes their embryos to develop. On the other hand, male numbers could grow immediately after offspring production had resumed because they cost little to produce. For this reason, sexuparae in the 1G + 1G treatment group invested most of their resources in males during the pause in reproduction. The number of viviparous aphids in the 1G + 1G and 1G + 1R treatment groups was significantly smaller than in the 1G treatment group, suggesting that in the paired treatment groups, sexuparae may have shifted resources that should have been invested in viviparous aphids to males or oviparous females to fulfill their sex allocation strategy. On the other hand, due to the lower cost required for male embryo development, sexuparae in the 1G + 1G treatment group invested most of their resources in males during the suspension of reproduction, so that the number of males could grow immediately after resuming reproduction. We hypothesize that during a moratorium on parturition, green clone sexuparae determine their subsequent sex investment strategy based on the type and density of clones in their vicinity. Sexuparae may adopt different competitive strategies when faced with different targets, ultimately leading to different offspring sex allocation patterns. This differentiation strategy might be an adaptive response to resource allocation and population optimization, reflecting the biological diversity and flexibility of *A. pisum* survival strategies in complex ecosystems, a phenomenon never reported before. It merits attention that *A. pisum* sexuparae are classified into three distinct categories predicated on the nature of their male progeny: winged males, wingless males, and both [51]. The sexuparae utilized in this study exclusively yielded winged male offspring. The competitive dynamics between other types and diverse non-self clones warrant further exploration.

In this study, three additional treatments were also established, 1G (0 ng JH), 1G + 20Gov (0 ng JH), and 1G + 20Rov (0 ng JH). In 1G (0 ng JH), the progeny sex allocation was similar to that in 1G, as no exogenous JH III was added. The G3 sex allocation in 1G + 20Gov (0 ng JH) and 1G + 20Rov (0 ng JH) was similar to that in 1G + 1G and 1G + 1R, respectively. Therefore, we hypothesize that *A. pisum* has developed a stable and effective strategy for sex allocation during long-term evolution. JH III significantly affects sex allocation in *A. pisum* sexuparae. The phenotypic differentiation of aphids is related to the titer of JH III, and a reduction in the JH III titer could promote male differentiation [27,52]. Under experimental conditions devoid of exogenous JH III supplementation, *A. pisum* sexuparae exhibited a spectrum of alterations in JH III titers six days post parturition onset, leading to a diverse range of variances in the sexual allocation of the ensuing G3 generation. Therefore, the *A. pisum* sexupara sex ratio regulation strategy identified in this study was achieved by regulating the JH III titer. The JH III titers on day 6 were 0.91 ng/aphid in the 1G treatment group and 0.37 ng/aphid in the 1G + 20Gov (0 ng JH) treatment group, in which the number of male aphids increased significantly, in accordance with the previous findings [24]. In the 1G + 20Rov (0 ng JH) treatment group, the sexupara JH III titer was 0.56 ng/aphid, and the number of oviparous female offspring was significantly higher than in the 1G (0 ng JH) treatment group. Subsequently to the administration of exogenous JH III, there was a notable increase in the population of viviparous females and a corresponding reduction in the number of sexual aphids across all treatment groups. Furthermore, the post-supplementation sex allocation pattern closely resembled that observed in the 1G treatment group. In other words, the artificial supplementation of JH III interfered with sexupara sex allocation. In future work, we intend to investigate the molecular mechanism through which JH III regulates sex allocation in *A. pisum*. Aphid offspring sex ratio is controlled by the sexuparae rather than sex chromosomes, as in many hymenopteran insects [53,54]. Under different competitive states, *A. pisum* may optimize sexual investment by regulating the JH III titer to ensure optimal reproductive strategies.

## 5. Conclusions

*A. pisum* sexuparae have the adaptive capability to modulate the sex allocation of their progeny based on the nature of neighboring competitor clones, demonstrating a range of sex allocation mechanisms. In this intricate biological process, JH III plays an integral and pivotal role.

## Data Availability

The original contributions presented in the study are included in the article, further inquiries can be directed to the corresponding author.
